# Intravenous injection versus transhepatic intracholecystic injection of indocyanine green (ICG) to outline biliary tree during laparoscopic cholecystectomy

**DOI:** 10.1186/s12893-024-02612-y

**Published:** 2024-10-25

**Authors:** Hesham A. Elmeligy, Hend F. Hassan, Moshira S. Amer, Yousra Ossama, Mohamed A. Maher, Ahmed M. Azzam, Mahmoud Rady

**Affiliations:** 1 General Surgery Department, Theodor Bilharz Research Institute (TBRI), Giza, Egypt; 2 Anaesthesiology Department, Theodor Bilharz Research Institute (TBRI), Giza, Egypt; 3 Pathology Department, October 6 University, Giza, Egypt; 4 Environmental Research Department, Theodor Bilharz Research Institute (TBRI), Giza, Egypt

**Keywords:** Common bile duct injury, Intracholecystic, Fluorescence cholangiography, Indocyanine green, Laparoscopic cholecystectomy

## Abstract

**Background:**

To potentially lessen injuries and associated complications, fluorescence cholangiography has been suggested as a technique for enhancing the visualization and identification of extrahepatic biliary anatomy. The most popular way to administer indocyanine green (ICG) is intravenously, as there is currently little data on ICG injections directly into the gallbladder. In order to visualize extrahepatic biliary anatomy during laparoscopic cholecystectomy (LC), we compared the two different ICG administration techniques. We also examined variations in visualization time, as well as the effectiveness, benefits, and drawbacks of each modality.

**Methods:**

In this prospective randomized clinical study, 60 consecutive adult patients with chronic and acute gallbladder disease were included. Our study conducted from 2022 to 2024 in Surgical Department of Theodor Bilharz Research Institute. Thirty patients underwent LC with intravenous ICG administration (IV-ICG), thirty patients received a direct injection of gallbladder through transhepatic ICG (IC-ICG) and Preoperative, intraoperative, and postoperative patient data were examined.

**Results:**

In terms of their perioperative and demographic features, the groups were similar. Without a statistically significant difference, the IV-ICG group’s total operating time was less than that of the IC-ICG group (p 0.140). Compared to the transhepatic IC-ICG method, IV-ICG was more accurate in identifying the duodenum and the common hepatic duct (*p* = 0.029 and *p* = 0.016, respectively). In the transhepatic IC-ICG and IV-ICG groups, the cystic duct could be identified prior to dissection in 66.6% and 73.3% of cases, respectively, and this increased to 86.6% and 93.3% following dissection. In the transhepatic IC-ICG group, the common bile duct was visible in 93.3% of cases; in the IV-ICG group, it was visible in 90% of cases. Two cases in the IC-ICG group and every case following IV-ICG administration had liver fluorescence (6.6% versus 100%; *p* < 0.001).

**Conclusion:**

The current study shows that for both administration methods, ICG-fluorescence cholangiography can be useful in identifying the extrahepatic biliary anatomy during Calot’s triangle dissection. By avoiding hepatic fluorescence, the transhepatic IC-ICG route can increase the bile duct-to-liver contrast with less expense and no risk of hypersensitivity reactions than the intravenous ICG injection method. We recommend to use both techniques in case of acute cholecystitis with cystic duct obstruction. In cases of liver cirrhosis, we recommend transhepatic IC-ICG as IV-ICG is limited.

## Introduction

The incidence of iatrogenic main bile duct lesions has increased significantly since laparoscopic cholecystectomy (LC) became widely used; incidences have been reported to range from 0.2 to 1.5% in previous studies [1–3]. Bile duct injuries (BDI) are still a major concern during LC, despite being on the decline with the application of critical view of safety (CVS) in the dissection of the elements that define Calot’s triangle. These are a potentially fatal consequence and one of the most common reasons for postoperative morbidity; they are also linked to longer hospital stays, higher medical expenses, or the need for additional procedures for treatment. The severity of the inflammatory process in acute cholecystitis (AC), fibro-sclerosing remodeling linked to chronic inflammation, and the anatomic variability of the bile duct—all of which increase the risk of converting to open surgery—are the primary risk factors for BDI [4].

The most common reason for switching from laparoscopy to laparotomy is postoperative adhesions [5]. Clinical trials thus show that many surgeons prefer to postpone LC in AC until remission of local inflammatory phenomena, even though this is advised in current practice protocols, in order to prevent iatrogenic damage to the main bile duct [6, 7]. The prevention of main bile duct lesions is a key focus of the new safe LC surgery protocols [1, 2]. An emerging technique that may improve visualization of the extra biliary structures is the ICG-assisted near-infrared cholangiogram (NIRC). Preventing injuries and ensuring safe laparoscopic cholecystectomy requires precise techniques, a thorough understanding of the local anatomy, and appropriate exposure of the extra hepatic biliary structures (3).

When dissection is difficult or there is a suspicion of main bile duct stones, intraoperative cholangiography (IOC) can be very helpful. However, there are a number of drawbacks, such as the need for portable radiology equipment, specialized staff, radiation, longer recovery times, and higher expenses. Regular IOC may not always be able to stop a BDI from happening, but it may help detect one at the time of surgery. Furthermore, IOC may raise the risk of BDI by necessitating the injection of contrast material into the bile duct [7].

The benefits of ICG NIRC were initially shown by Ishizawa et al. in 2009 when they administered it intravenously prior to an open cholecystectomy and via direct injection into the bile duct [8]. This innovative method was very alluring, especially considering that the most recent laparoscopic systems now have software features that enable NIR image acquisition and information overlay over white light images. As a result, more information can be obtained without lengthening the operating period or altering the operating time sequence [9].

ICG is a dye that is currently used in a number of surgical and medical specialties, including cardiology, ophthalmology, and abdominal surgery. After hepatic extraction, the dye is nearly entirely eliminated into the bile after binding with circulating albumins and lipoproteins. Its half-life in the blood is three to five minutes [10]. Direct injection into the gallbladder is an alternative in biliary surgery. The NIR emission of the ICG molecule has a peak in its spectrum between 810 and 830 nm, which allows it to conveniently avoid endogenous interferences from body proteins and water. It is safe for human use, but because it is crystallized with iodized salts, it should not be used by people who are allergic to iodine [11].

This study aims to compare the safety, efficacy, feasibility, and accuracy of intravenous and transhepatic IC-ICG injection of Near Infra-Red ICG fluorescent in defining the biliary anatomy during LC, which may reduce the duration of the procedure, the conversion rate, and the incidence of biliary lesions, as well as to evaluate the impact on liver and kidney function.

## Materials and methods

After informed consent, 60 patients with acute or chronic cholecystitis scheduled for NIR-ICG fluorescent cholangiography during LC were consecutively included in the study. ICG was administered intravenously or intracholecystic injection during surgery. This prospective randomized comparative was evaluated based mainly on the clear anatomical delineation of the gall bladder, cystic duct, hepatic ducts and common bile duct. This study had been conducted from 2022 to 2024 with the approval of the institutional ethics committee.

Inclusion criteria included patients from 16 to 80 years old, patients with gall bladder pathology (cholecystitis, gall bladder polyp) and patients fit for laparoscopic cholecystectomy.

Exclusion criteria included patients with contraindication for laparoscopic cholecystectomy (for example patients had significant pulmonary or cardiac problems or severe renal insufficiency), intraoperative dye spillage during dissection of the gallbladder, obstructive jaundice, proven or suspected allergies to ICG, pregnancy or lactation.

The patients were classified into 2 groups, group A patients (*n* = 30) were chosen for intravenous injection of ICG while group B patients (*n* = 30) were chosen for transhepatic intracholecystic injection of ICG during cholecystectomy.

To randomise, a sealed opaque envelope was chosen based on a computer-generated random sequence. Surgeons with extensive experience in biliary surgery performed all laparoscopic procedures. The patient’s age, sex, BMI, comorbidities, and cholecystectomy indication were among their characteristics. Perioperative data included the percentage of non-elective versus elective procedures, the length of time from skin incision to skin closure during the procedure, the location of intraoperative drains, the estimated blood loss (EBL), and the necessity of switching from a laparoscopic to an open approach.

Of note, patients undergoing a concomitant procedure during LC (i.e., esophagogastroduodenoscopy, liver biopsy, lap gastric band removal, etc.) were excluded from the operative time analysis.

**Fig. 1:**
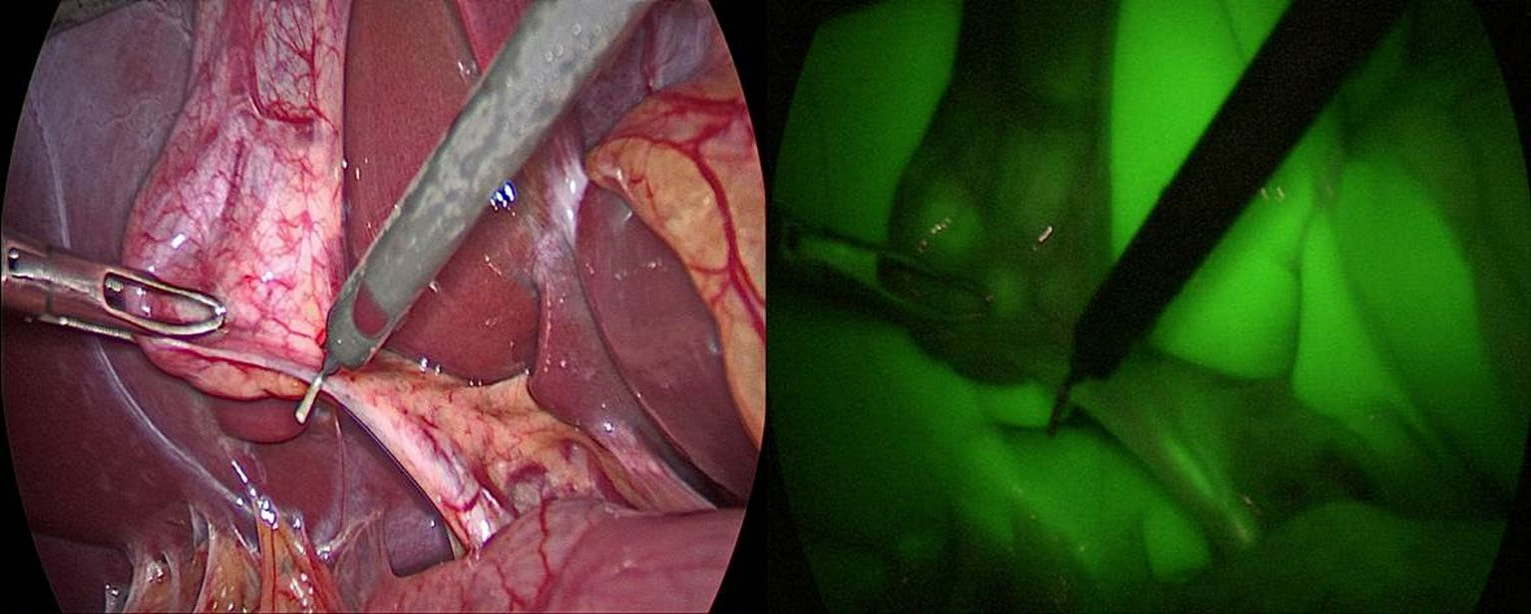
FC prior to dissection of Calot’s triangle

**Fig. 2:**
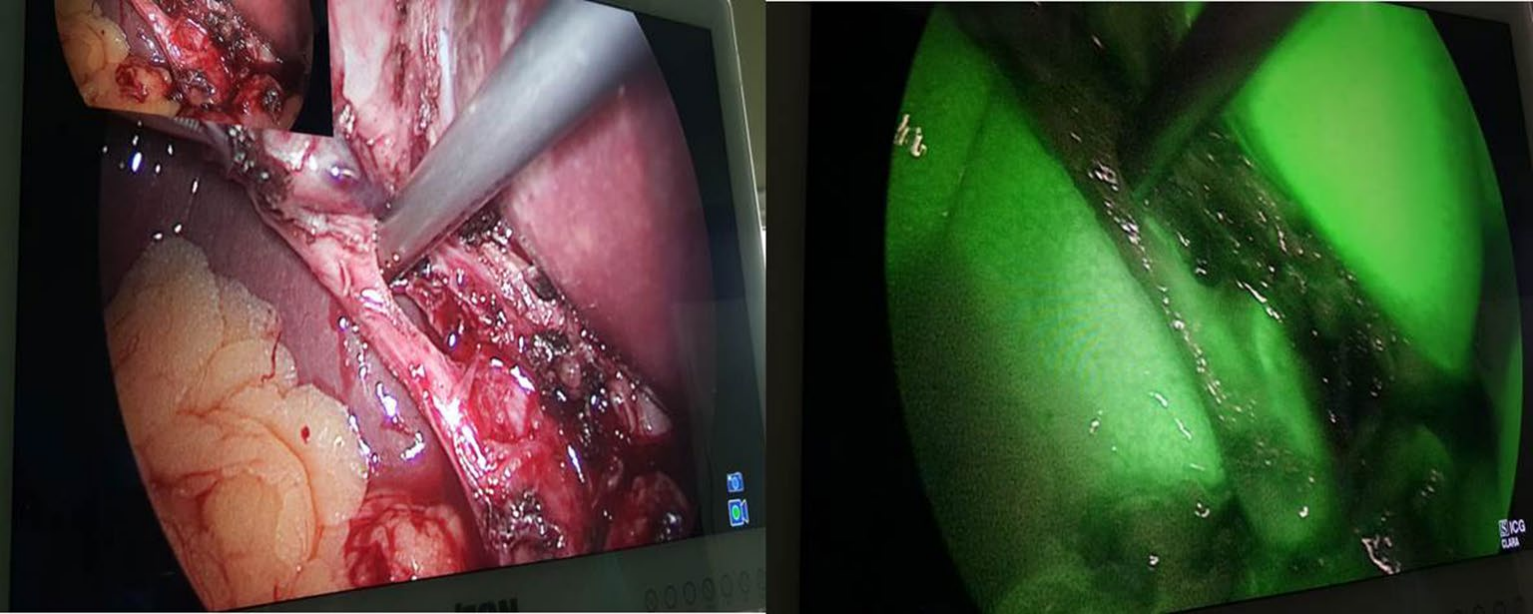
FC after to dissection of Calot’s triangle

In group A (IV-ICG) intravenous administration of ICG was done in 30 patients, images were taken before and after dissection of calot’s triangle (Figs. 1 and 2).

**Fig. 3:**
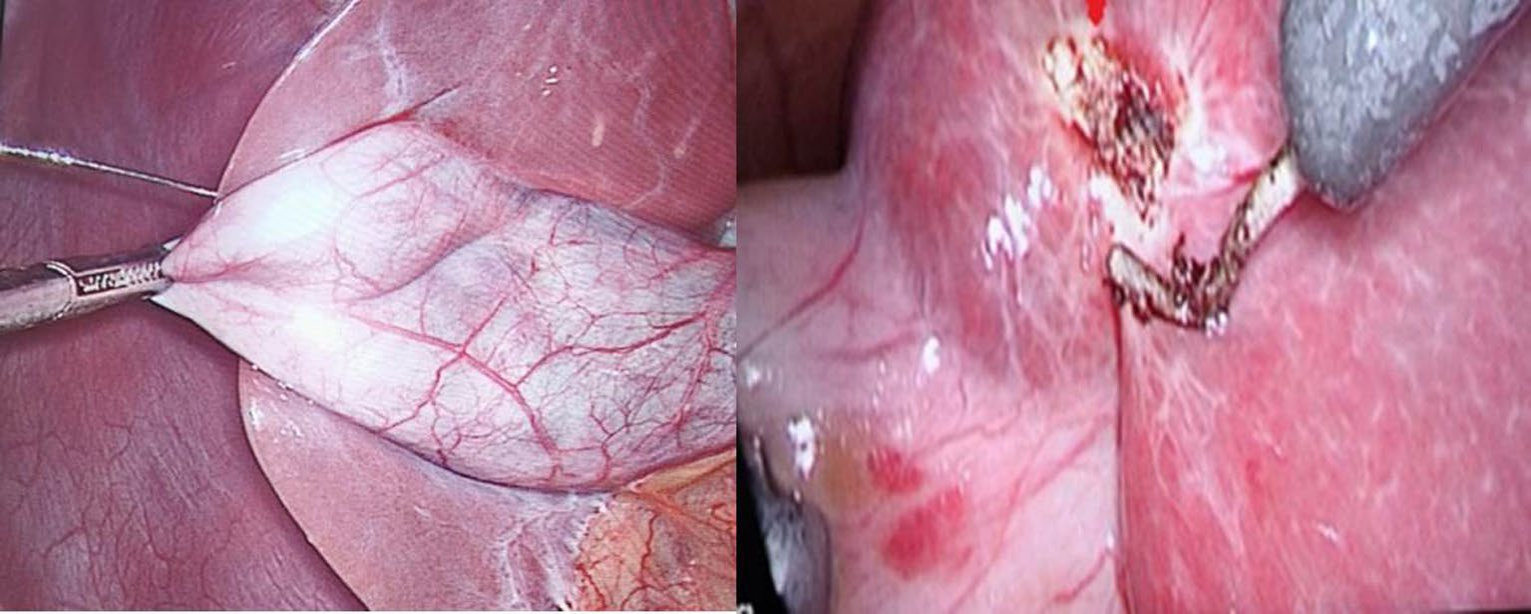
Technique of intracholecystic ICG njection

**Fig. 4:**
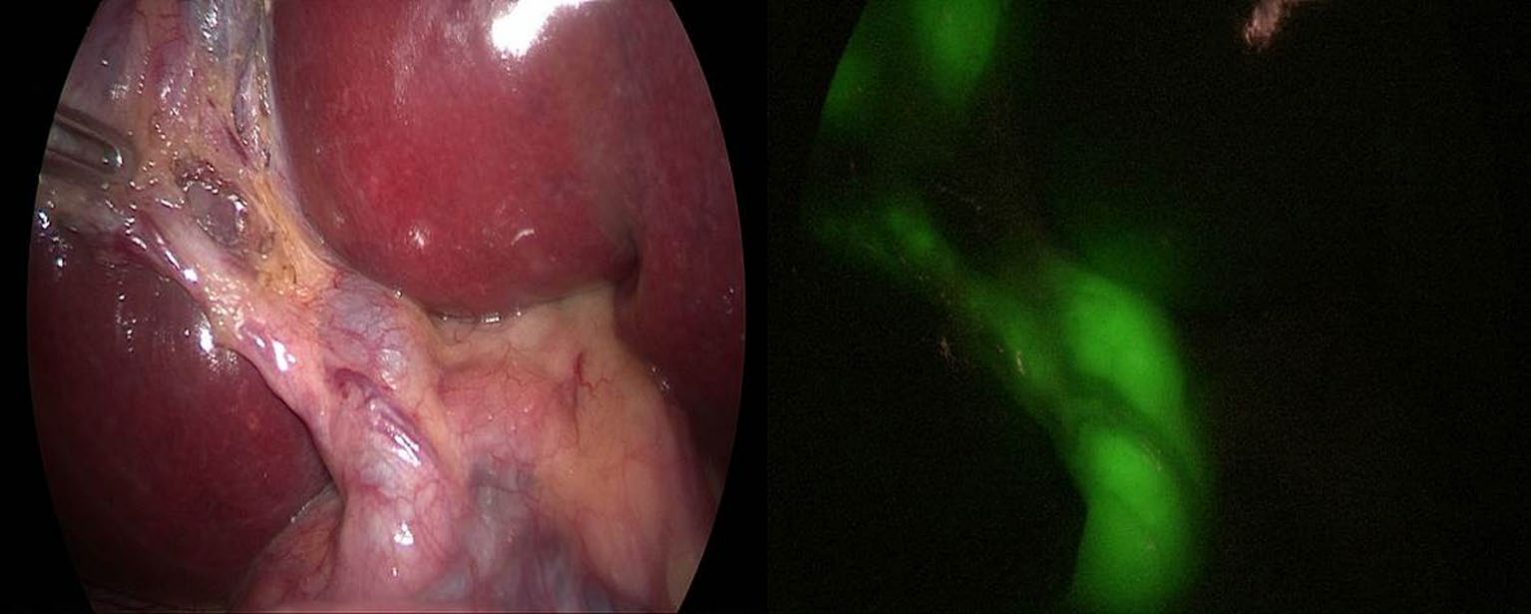
FC prior to dissection of Calot’s triangle

In group B (IC-ICG): Transhepatic intracholecystic injection of ICG was done in 30 patients. 1.25 mg of ICG powder from a 25 mg ICG vial were separated under aseptic conditions and dissolved in 3 ml of saline, and the concentration was roughly 0.04 mg after each 1 ml was diluted by 9 ml of saline. In order to prevent dye leakage that might result in false FC results, a veress needle was inserted through the abdominal wall and into the gall bladder fundus through the liver parenchyma (transhepatic), then puncture site was cauterized (Fig. 3). Then the image was taken after dissection of calot’s triangle (Fig. 4).

Usually, the gallbladder was removed through the umbilical or epigastric port. From the moment the first incision was made until the last wound was closed, the operating time was tracked. After excision of the gall bladder, all sent to pathology department for histopathology examination to exclude malignancy, confirm cholecystitis, and detect type of stones. On the first postoperative day, patients were sent home. An analysis was conducted on the following factors: operation time, dye spillage due to technique due to injection, cystic duct, common hepatic duct, common bile duct delineation, drain insertion, postoperative hospital stays, complications, and biliary injury.

### Statistical methods

Student’s t test was used to compare the data with a normal distribution and the continuous variable data, which were provided as mean ± standard deviation. When appropriate, the Fisher’s exact test or the Chi-square test was used to compare categorical variables, and logistic regression was employed for univariate analysis. SPSS version 20.0 was used for the statistical analysis (SPSS Inc., Chicago, IL, USA). P values less than 0.05 were regarded as statistically significant.

## Results

After meeting the study’s inclusion requirements, a total of 60 patients had laparoscopic cholecystectomy procedures. In group A, 30 patients (50%) underwent FC via IV ICG, and in group B, 30 patients (50%) underwent transhepatic intracholecystic FC. In group A, the mean age ranged from 19 to 80 years, while in group B, it ranged from 18 to 75 years (*p* = 0.1922). In group A, there were 73.33% more female patients than in group B (*p* = 0.1757). Group A’s average BMI was 31.92 kg/m2, whereas group B’s average BMI was 28.48 kg/m2 (*p* = 0.1632). A range of indications for cholecystectomy were given to the patients: GB polyps in group A versus group B: 3.3% vs. 6.6%, biliary colic in 80% vs. 73.3%, acute cholecystitis in 13.3% vs. 16.6%, and gallstone pancreatitis in a single patient in group A (*p* = 0.2532). Table 1 displays the demographic information and indications for all patients undergoing laparoscopic cholecystectomy collectively.

**Table 1:** Demographic data

		Group A (IV-ICG) (n = 30)	Group B (IC-ICG) (n = 30)	*P* value
Sex (n, %)	Male	8 (26.6%)	6 (20%)	0.4757
	Female	22 (73.3%)	24 (80%)	
Age (Mean ± SD)		40.5 ± 11.5	43.2 ± 12.9	0.8922
BMI (Mean ± SD)		31.92 ± 3.94	28.48 ± 4.41	0.1632
Indications of cholecystectomy (n, %)	Biliary colics	25 (83.3%)	26 (86.6%)	0.2532
	Acute attack	3 (10%)	2 (6.6%)	
	GB polyp	1 (3.3%)	2 (6.6%)	
	GB pancreatitis	1 (3.3%)	0	

Table 2 presents an overview of ICG’s capacity to distinguish the anatomy of the bile duct based on the mode of administration. In comparison to the transhepatic IC-ICG method, IV-ICG was more accurate in identifying the duodenum and the common hepatic duct (CHD) (*p* = 0.029 and *p* = 0.016, respectively). However, there were no significant difference in the two groups’ visual perception of the gallbladder, the common bile duct (CBD), the cystic duct (CD) before and after dissection, and the CD-CHD confluence. Specifically, ICG-fluorescence was able to visualize the gallbladder in 90% of cases in the IV-ICG group and 100% of cases in the transhepatic IC-ICG group because IV-ICG cannot visualize the gall bladder in the 3 cases of acute cholecystitis. In the IV-ICG and transhepatic IC-ICG groups, the cystic duct could be identified prior to dissection in 66.6% and 93.3% of cases, respectively, and this increased to 88.6% and 93.3% following dissection. All cases of acute cholecystitis in both groups could not visualize the CD. In 93.3% of cases in the transhepatic IC-ICG group and 90% of cases in the IV-ICG group, the common bile duct (CBD) could be clearly seen. In the transhepatic IC-ICG group, liver fluorescence was observed in 2 cases, while in the IV-ICG administration group, it was present in all cases (6.6% versus 100%; *p* < 0.0001). One patient in the transhepatic IC-ICG group, who also happened to be one of the two with acute cholecystitis, had no visible biliary structures within the group transhepatic IC-ICG.

**Table 2:** Comparison of ICG cholangiography before dissection in delineation of extrahepatic biliary tree in both groups

		Group A (IV-ICG)(n = 30)	Group B (IC-ICG)(n = 30)	*P* value
Liver		30 (100%)	2 (6.6%)	< 0.001
Gall bladder (n, %)	Before	27 (90%)	30 (100%)	0.752
	After	27 (90%)	30 (100%)	
Cystic duct (n, %)	Before	20 (66.6%)	22 (73.3%)	0.877
	After	26 (86.6%)	28 (93.3%)	
Cystic-hepatic junction (n, %)	Before	16 (53.3%)	14 (46.6%)	0.896
	After	28 (93.3%)	26 (86.6%)	
Common bile duct (n, %)	Before	20 (66.6%)	15 (50%)	0.306
	After	28 (93.3%)	27 (90%)	
Common hepatic duct (n, %)	Before	16 (53.3%)	14 (46.6%)	0.029
	After	28 (93.3%)	16 (53.3%)	
Duodenum (n, %)	Before	16 (53.3%)	10 (33.3%)	0.016
	After	28 (93.3%)	14 (46.6%)	

In spite of a pre-injection negative test, one patient in the IV-ICG group experienced delayed hypersensitivity. In neither group was there a record of bile duct injury. Table 3 shows that the IV-ICG group’s total operating time was significantly less than that of the transhepatic IC-ICG group, with no discernible difference (*p* = 0.140). In the transhepatic IC-ICG group, bile/ICG spillage due to injection was nil due to our new innovated transhepatic technique. Regarding postoperative pain, there was no discernible difference between the two groups (*p* = 0.327).

**Table 3:** Comparison of other intraoperative and postoperative outcomes in both groups

	Group A (IV-ICG) (*n* = 30)	Group B (IC-ICG) (*n* = 30)	*P* value
Duration of surgery (min)	32 ± 16	38 ± 23	0.140
Length of stay (days)	1 (0–2)	1 (0–2)	-
Hypersensitivity (n,%)	1 (3.3%)	0 (0)	0.776
Intra-operative complications (n,%)	0 (0)	0 (0)	-
Postoperative complications (n,%)	0 (0)	0 (0)	-
Postoperative pain score	6.2 ± 2.6	5.7 ± 2.3	0.327

## Discussion

There have been few studies to date that actively compare these two distinct approaches, but intraoperative fluorescence cholangiography can represent a quick, non-invasive, technically straightforward modality for achieving real-time cholangiographic images, allowing for administration. The use of intravenous ICG fluorescence cholangiography is becoming more and more supported by data [12]. When ICG is administered systemically, it accumulates in hepatocytes and is then eliminated through bile. However, the reduction in the signal-to-background ratio, which results in a high interference of the liver brightness with the visualization of extrahepatic biliary structures, is caused by ICG passing through the liver first [13].

Moreover, biliary excretion can affect the fluorescence output and is unpredictable, particularly in patients with liver dysfunction. While there is no denying this interference, there are a few tactics that could help optimize bile duct delineation. To get sufficient imaging, the timing of the intraoperative intravenous injection and the right ICG dosage are essential. After experimenting with various intravenous ICG dosages, Zarrinpar et al. determined that the ideal dosage is 0.25 mg/kg 45 min prior to surgery [14].

To visualize the extrahepatic bile ducts with little interference with liver brightness we used an even lower dose (0.01 mg/kg) 20 min prior to surgery. Conversely, Verbeek et al. discovered that the best combination for achieving minimal hepatic background fluorescence was an intermediate dose (10 mg) and delayed timing to surgery (24 h). They did this by experimenting with different ICG concentrations (5, 10, 20 mg) and administration timings (30 min versus 24 h preoperatively) [15]. To standardize intravenous ICG cholangiography, the timing and dosage concerns still need to be thoroughly evaluated through carefully planned studies.

Because ICG is directly injected into the gallbladder, its hepatic flow is avoided, which accounts for the background liver fluorescence and potentially enhances the visualization of the biliary tree. In this investigation, direct transhepatic IC-ICG injection proved beneficial for quickly identifying extrahepatic biliary structures at low ICG dosages that were safe and comparable to intravenous (IV) methods. The first report of direct ICG injection into the gallbladder in 11 patients was made by Graves et al. Nevertheless, there was no control group for the study’s small, single-arm patient population to compare results with [16]. The authors came to the conclusion that this approach was helpful in immediately defining extrahepatic biliary structures and in making the plane of dissection between the hepatic bed and gallbladder clearer. Similarly, in a larger group of patients undergoing laparoscopic cholecystectomy, Liu et al. administered ICG via the same injection route [13]. Two methods were used to accomplish this: in 18 patients, a pre-existing percutaneous transhepatic gallbladder catheter was used, and in 28 subjects, an intraoperative percutaneous gallbladder needle puncture was used. For the current investigation, we advise transhepatic IC-ICG injection along with entry site cauterization to prevent dye leakage that could produce inaccurate results.

The authors came to the conclusion that cholecystocholangiography was more helpful in identifying bile ducts than white light alone, particularly when gallbladder inflammation was present. When Skrabec et al. compared laparoscopic cholecystectomy with IC-ICG administration to the conventional method without ICG-Cholangiography, they observed no statistically significant differences in perioperative complications or operative time between the groups. However, they did highlight the value of IC-ICG in delineating the biliary tree [17]. 23 patients received direct gallbladder ICG injections in a prospective single-arm study by Cardenas et al., and all of them were able to show an optimal critical view of safety [18]. With 12 patients in each group, Shibata et al. [19] compared the differences between intravenous ICG administration and intrabiliary administration. In contrast, there were three distinct methods used to administer ICG to patients in the intrabiliary group: eight patients underwent transhepatic gallbladder drainage, one patient underwent endoscopic nasobiliary drainage, and three patients received direct gallbladder injection. Castagneto-Gissey et al. carried out a prospective case-control study that included 35 adult patients having acute or chronic cholecystitis. 18 patients had LC with intravenous ICG, and 17 patients had transcholecystic ICG injection. The intravenous group had a significantly shorter operative time in comparison to the intracholecystic group (*p* = 0.017). It delineated the common hepatic duct and the duodenum in comparison to intracholecystic group (*p* = 0.041 and *p* = 0.009, respectively). The visualization of the predissected cystic duct was clearer in the IC-ICG group (76.5%) than in the IV-ICG group (66.7%) increased after dissection to 88.2% and 83.3%. Visualization of CBD was 76.5% in the IC-ICG group and 77.8% in the IV-ICG group. Fluorescence of the liver was present in all cases after IV-ICG injection and in one case in the IC-ICG group (100% versus 5.8%; *p* < 0.0001). They concluded that both techniques can help delineate the extrahepatic biliary tree while dissecting the Calot’s triangle. The IC-ICG has a better signal than the IV-ICG by avoiding the hepatic fluorescence background ratio [20].

We cannot definitively conclude that one modality is better than another using the non-standardized intrabiliary approach. Dip et al. conducted the sole randomized controlled trial that has been published in the literature. They compared the effectiveness of near-infrared fluorescent cholangiography with white light alone in terms of bile duct visualization, and they discovered that fluorescence was significantly better at highlighting biliary structures prior to dissection than the latter group [21].

Both techniques showed that fluorescence cholangiography can be a useful tool for defining the extrahepatic biliary anatomy and making it easier to identify the components of the Calot’s triangle. By avoiding hepatic fluorescence and improving the bile duct-to-liver contrast, direct IC-ICG injection can offer a clearer view of the gallbladder and bile ducts than IV-ICG administration. Indeed, there was a significant difference in liver fluorescence between the IC-ICG and IV-ICG groups (6.6% versus 100%; *p* < 0.001). The incorrect administration of ICG in the gallbladder wall rather than its lumen was the cause of the two cases of hepatic fluorescence that happened in the IC-ICG group. ICG did not need to be given before surgery in order to perform an intraoperative real-time cholangiography using the IC-ICG technique. Because the ICG did not need to be surgically injected, the IV-ICG group’s total operative time was less than that of the IC-ICG group (*p* = 0.140) without a statistically significant difference.

In cases of acute cholecystitis, there were some limitations in both techniques. The inability of ICG to flow downstream towards the CBD using transhepatic IC-ICG modality prevented visualization of the biliary structures except the GB, also there was inability to visualize the GB and CD in IV-ICG modality so we recommend using the both approaches in cases of acute cholecystitis. When compared to the IC-ICG method, IV-ICG performed better at differentiating between the duodenum and the CHD (*p* = 0.029 and *p* = 0.016, respectively). On the other hand, the ability to visualize the gallbladder, CyD pre- and post-dissection, the CBD, and the CyD-CBD confluence were similar between the two groups.

## Conclusion

The current study shows that after both administration methods, ICG-fluorescence cholangiography can be useful in identifying the extrahepatic biliary anatomy during the dissection of Calot’s triangle. By avoiding hepatic fluorescence, the transhepatic IC-ICG route may offer a better signal-to-background ratio than intravenous ICG administration. This will increase the contrast between the bile duct and liver. However, an improved visualization of the proximal part of the extrahepatic biliary tree is favored by the intravenous route. Because there was no risk of hypersensitivity and the dose was lower, the transhepatic IC-ICG route was less expensive than the IV route. In cases of liver cirrhosis, we recommend transhepatic IC-ICG as IV-ICG is limited. We recommend using both techniques in case of acute cholecystitis with cystic duct obstruction. Through enhanced visualization of the biliary tree, these methods may help reduce the chance of bile duct injury during laparoscopic cholecystectomy. The Trial registration: ClinicalTrials.gov Identifier: NCT06629415.

## Data Availability

The datasets used and/or analyzed during the current study are available from the corresponding author on reasonable request.
